# P32 A case of disseminated pneumococcal infection with musculoskeletal manifestations

**DOI:** 10.1093/rap/rkae117.063

**Published:** 2024-11-05

**Authors:** James Carroll, Nadia Ahmad

**Affiliations:** Basingstoke and North Hampshire Hospital, Basingstoke, United Kingdom; Basingstoke and North Hampshire Hospital, Basingstoke, United Kingdom

## Abstract

**Introduction:**

Septic arthritis should be the first consideration in any patient presenting with a hot, swollen joint. Infection may affect one or more joints, and bacteria typically enter the joint through direct inoculation, haematogenous seeding, or spread from adjacent soft tissue or bone infection. Staphylococcus aureus is the most common causative organism. Streptococcus pneumoniae is an uncommon cause of septic arthritis, accounting for approximately 5% of cases. We present the case of a 78-year-old male with no significant medical history who presented with a polyarticular pattern of joint inflammation related to disseminated pneumococcal infection.

**Case description:**

The patient presented with a one week history of gradual onset pain and swelling in the right knee and right wrist. He was previously well and remained physically active. He was a non-smoker, drank moderate alcohol and had no history of intravenous drug use. He had no current or preceding infective symptoms, including respiratory tract symptoms, nor history of trauma or recent foreign travel.

Examination revealed a warm right knee effusion with restricted range of movement. There was swelling of both wrists (right more than left), both second metacarpophalangeal joints and the left ankle. There were no heart murmurs or peripheral stigmata of endocarditis. He was afebrile and haemodynamically stable.

Admission blood tests showed WBC 21 x10^9^/L, neutrophils 18 x10^9^/L, CRP 369 mg/L, urate 452 µmol/L and procalcitonin 3.84 µg/L. Rheumatoid factor was 25.3 IU/mL with negative anti-CCP. Knee X-ray showed significant medial compartment osteoarthritis. Hand and wrist X-rays were unremarkable.

Synovial fluid aspirate from the right knee grew Streptococcus pneumoniae and calcium pyrophosphate crystals were also seen on polarised light microscopy. Blood cultures were positive for the same organism.

The patient underwent arthroscopic washout of the knee and was treated with colchicine and intravenous amoxicillin. He was taken to theatre for a right wrist washout, however the wrist was found to be clean and dry intraoperatively. He had two further washouts of the right knee and repeat intraoperative samples and blood cultures were negative.

Additional screening for HIV, syphilis and myeloma was negative. Transthoracic echocardiogram showed no vegetations. CT chest showed basal atelectasis but no focal consolidation. Left hand and wrist MRI showed extensor compartment tenosynovitis and second metacarpophalangeal joint synovitis.

His antibiotics were switched to intravenous ceftriaxone and oral doxycycline. He clinically improved and was discharged with a plan to complete a 6-week course with outpatient review.

**Discussion:**

Streptococcus pneumoniae is an uncommon cause of septic arthritis, responsible for approximately 5% of all cases, but more often causes polyarticular infection than other organisms. This may be related to higher rates of bacteraemia in patients with pneumococcal septic arthritis. In one review of 190 cases of pneumococcal septic arthritis, polyarticular infection was present in 36% of patients and 72% had concomitant bacteraemia (compared with rates of approximately 20% polyarticular infection and 30-50% bacteraemia for septic arthritis overall). The joints most commonly affected are the knee, followed by the hip, shoulder, ankle and elbow. The original focus of infection is most commonly pneumonia or meningitis, but up to 50% of patients have no identifiable extra-articular source of infection.

Pneumococcal septic arthritis more commonly affects patients with predisposing risk factors, such as rheumatoid arthritis, osteoarthritis, joint prosthesis, alcoholism, myeloma and other malignancies, diabetes and immunosuppression. Our patient had no specific predisposing medical conditions other than knee osteoarthritis, and his age was likely to be his main risk factor. It is also important to note that septic arthritis in the elderly can present atypically, with insidious onset and absence of fever or other signs of systemic infection. This was the case in our patient, who despite bacteraemia and markedly elevated inflammatory markers, did not otherwise appear floridly septic or unwell.

Our patient presented with a polyarticular pattern of joint inflammation, although joint infection was only ultimately confirmed in the right knee. Also notable was the presence of calcium pyrophosphate crystals in the aspirate. One might theorise whether the infection might have triggered a reactive polyarthropathy or concurrent crystal arthritis. It is well recognised that septic arthritis and crystal arthritis can coexist in the same patient. The case also highlights the value in sampling multiple joints and taking blood cultures in such patients.

**Key learning points:**

• Polyarthritis does not exclude the possibility of septic arthritis

• Joint aspiration should be performed in all patients presenting with a hot, swollen joint

• In cases of polyarthritis, consideration should be given to aspiration of all affected joints and taking blood cultures, particularly in the elderly and patients with predisposing risk factors for septic arthritis

• Septic arthritis can present atypically in the elderly, with the absence of fever and other typical signs of systemic infection

